# Strategies for Enhanced Drug Delivery to the Central Nervous System

**DOI:** 10.4103/0250-474X.41446

**Published:** 2008

**Authors:** V. S. N. M. Dwibhashyam, A. N. Nagappa

**Affiliations:** Pharmacy Practice Department, 4th Floor, Shirdi Sai Baba Cancer Hospital, Manipal-576 104, India; 1 TherDose Pharma (P) Ltd., Plot No: 30-32, Survey No. 400, Prasanth Nagar, IE, Hyderabad-500 072, India

**Keywords:** CNS drug delivery, p-glycoprotein, nano particles, blood brain barrier

## Abstract

Treating central nervous system diseases is very challenging because of the presence of a variety of formidable obstacles that impede drug delivery. Physiological barriers like the blood-brain barrier and blood-cerebrospinal fluid barrier as well as various efflux transporter proteins make the entry of drugs into the central nervous system very difficult. The present review provides a brief account of the blood brain barrier, the P-glycoprotein efflux and various strategies for enhancing drug delivery to the central nervous system.

The brain is a delicate organ. The brain-microvascular endothelial cells of the blood-brain barrier protect this organ from exogenous substances. Certain efflux transporters such as P- glycoprotein also perform the same function. However, these protective barriers restrict the entry to the brain from the periphery of compounds that might be of therapeutic value in the treatment of fatal central nervous system (CNS) diseases, such as brain tumors, HIV encephalopathy, epilepsy, cerebrovascular disease and neurodegenerative disorders, and of other pathologies. Delivery of drugs to the brain is a challenging task because of the presence of efficient protective mechanisms. The existing conventional drug delivery systems have been inefficient in selectively targeting drugs to the CNS. In response to this, aggressive research efforts have focused on the development of new strategies to more effectively deliver drug molecules to the brain 1.

## THE BLOOD BRAIN BARRIER (BBB) AND P-GLYCOPROTEIN (P-gp)

**Fig. 1:**
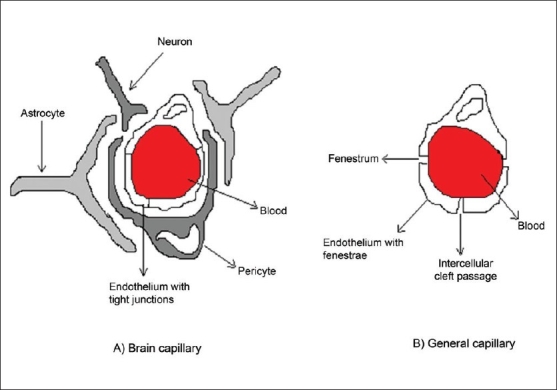
Schematic comparison between brain and general capillaries. The epithelium in brain capillaries are thickened with fat which acts as tight junctions and barrier for water soluble molecules in systemic circulation.

The BBB is one of the most challenging barriers in the body. It is created by the way the blood vessels in the brain are organized. Brain capillaries are different from capillaries of other parts of the body in that normal brain endothelia have fewer pinocytic vesicles, more mitochondria, no fenestrations and adjacent cells are maintained in close apposition by tight junctions 2–4. The shielding effect of the BBB is further strengthened by the presence of certain efflux transporters such as P-gp in the luminal membrane of the cerebral capillary endothelium. (Schematic depiction of comparison between brain capillaries and capillaries in general is given in fig. 1). P-gp is a brain microvascular endothelial cell protein, which possesses several essential pharmacological functions of drug portage and expulsion 5. It is present in high concentration on the apical surface of these endothelial cells. P-gp is an active drug efflux transporter protein. While P-gp is involved in protecting the brain exposure to a variety of pharmacologically active hydrophobic agents, it is an impediment to the treatment of various CNS diseases such as primary brain tumors and cerebral human immuno deficiency virus (HIV) infection 5. P-gp has affinity for a broad range of structurally unrelated large hydrophobic compounds including Vinca alkaloids, epipodophyllotoxins, anthracyclines, cyclosporine A, digoxin, and various HIV protease inhibitors 6–9. Various approaches are being tried to bypass P-gp efflux. Reversal agents such as R-verapamil, PSC 833 (cyclosporine analog), and biricodar, inhibit P-gp mediated drug transport and increase the influx of therapeutic agents they are co-administered with 10. Certain non-ionic surfactants like Tween-80 and Cremophor EL have also been found to have the reversal activity 11,12. However, most of these agents have been found to be pharmacologically active and elicit significant toxicity at doses required for P-gp inhibition.

## STRATEGIES FOR ENHANCED CNS DRUG DELIVERY

Various strategies have been studied to circumvent the multitude of barriers inhibiting brain penetration by therapeutic agents. These strategies generally fall into one or more of the following three broad categories viz., manipulating drugs, disrupting the BBB, and exploiting alternative routes for drug delivery

## ENHANCING BBB PERMEABILITY BY DRUG MANIPULATION

### Lipophilic analogs:

Drug penetration through the BBB is favored by lipophilicity. Because a drug's lipophilicity correlates so strongly with cerebrovascular permeability, hydrophobic analogs of small hydrophilic drugs ought to more readily penetrate the BBB. However, the drug molecule should have an optimum octanol-water partition coefficient with Log P value of approximately 1.5 to 2.5 to be efficacious when delivered via the circulatory system 13.

### Prodrugs:

A prodrug consists of a drug covalently attached to an unrelated chemical moiety that improves the drug's pharmacokinetic properties. The prodrug itself is inactive but becomes active when the attached moiety is cleaved *in vivo* by enzymatic or hydrolytic processes. To enhance a drug's penetration through the BBB, prodrugs are often designed by attaching chemical moieties that increase the drug's lipophilicity. The best example of this approach is the series of analogs of morphine. Morphine does not readily cross the BBB. Latentiation via acetylation of both hydroxyl groups yields the hallucinogenic heroin, which readily traverses the BBB, and subsequent hydrolytic cleavage of the acetyl groups yields high concentrations of morphine trapped in the brain due to its hydrophilicity 2.

### Chemical drug delivery systems (CDS):

The chemical delivery systems cross the BBB by smuggling compounds across as their lipophilic precursors. The CDS approach is a part of retro metabolic drug design approach. These are inactive chemical derivatives of a drug obtained by one or more chemical modifications that provide site-specific or site enhanced delivery through multi-step enzymatic and/or chemical transformations. They include two types of bio-removable moieties: a targeter (T) responsible for targeting, site-specificity, and lock-in; and modifier functions (F_1_-F_n_) that serve as lipophilisers, protect certain functions, or fine tune the necessary molecular properties to prevent premature, unwanted, metabolic conversions. Targeting is achieved by design: CDSs will undergo sequential metabolic conversions, disengaging the modifier functions and finally the targeter, after they fulfill their site- or organ-targeting role.

There are a wide variety of CDSs possible both theoretically and in practice. For convenience, the major CDSs can be divided into three classes. They are a) enzymatic physico-chemical CDSs that exploit site-specific traffic properties by sequential metabolic conversions resulting in altered transport properties. b) Site-specific enzyme activated CDSs that exploit specific enzymes found primarily, exclusively, or at higher activity at the site of action and c) Receptor-based CDSs that enhance selectivity and activity through transient, reversible binding to target receptors.

Brain targeting CDSs based on altered transport properties are the most studied ones, that are based on the idea that if a lipophilic compound that enters the brain is converted there into a lipid insoluble molecule, it will become ‘locked in’. Bodor *et al,* developed a creative approach to exploit the permeability characteristics of the BBB to achieve site-specific and/or sustained release of drugs to the brain 14–16. Targeting is assisted because the same conversion-taking place in the rest of the body accelerates elimination.

**Fig. 2:**
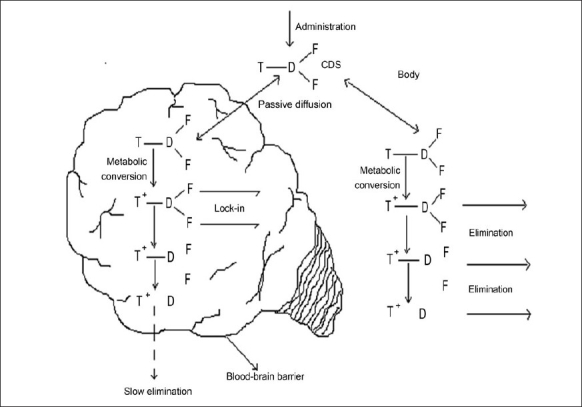
The lock-in mechanism for a brain chemical drug delivery system (CDDS). Lipid soluble drug after entering the brain gets converted into less lipid soluble drug by metabolic conversion. The formed metabolite gets entrapped in the brain, which ensures longer residence leading to longer duration of action and targeting action. T- Targeter moiety (lipophilic form); D- drug; F- modifier function; T^+^- Targeter moiety (hudrophilic form).

In principle, many targeter moieties are possible, but the one based on the 1,4-dihydrotrigonelline-Trigonelline system, where the lipophilic 1,4-dihydro form (T) is converted *in-vivo* to the hydrophilic quaternary form (T ^+^), proved the most useful. This conversion takes place easily everywhere in the body because it is closely related to the ubiquitous NADH-NAD ^+^ coenzyme system associated with cell respiration. Oxidation takes place with direct hydride transfer, without generating highly active or reactive radical intermediates, providing a non-toxic targeter system. The resulting T ^+^ is locked in the brain (fig. 2), but is easily eliminated from the body due to acquired positive charge, which enhances water solubility. After a relatively short time, the delivered drug D (as the inactive, locked-in T ^+^ --D) will be present essentially only in the brain, providing sustained, brain-specific release of the active drug. These systems have been tried for a wide range of drug classes such as steroid hormones, antiinfective agents, anticancer agents, and anti retroviral agents among these, oestradiol-CDS is the most advanced and is currently undergoing Phase 1 and 2 clinical trials.

### Molecular packaging:

Delivering the peptides like enkephalin, TRH (thyrotropin-releasing hormone), and Kyotorphin analogs through the BBB is an even more complex problem because they can be rapidly inactivated by ubiquitous peptidases 17–20. Three issues are to be solved simultaneously to enhance penetration through BBB. They are, to enhance passive transport by increasing the lipophilicity, assure enzymatic stability to prevent premature degradation, and exploit the lock-in mechanism to provide targeting. This complex approach is known as molecular packaging strategy, where the peptide unit is part of a bulky molecule, dominated by groups that direct BBB penetration and prevent recognition by peptidases. In general, a brain targeter packaged peptide delivery system contains a red-ox targeter (T), a spacer function (S), consisting of strategically used amino acids to ensure timely removal of the charged targeter from the peptide, the peptide itself (P) and a bulky lipophilic moiety (L) attached through an ester bond or sometimes through a C-terminal adjuster (A) at the carboxy terminal to enhance lipid solubility and to disguise the peptide nature of the molecule.

The first successful delivery with a package was for Tyr-D-Ala-Gly-Phe-D-Leu (DADLE), an analogue of leucine enkephalin, a naturally occurring linear pentapeptide (Tyr-Gly-Gly-Phe-Leu) that binds to opioid receptors. A similar strategy was used to deliver a thyrotropin-releasing hormone (TRH) analogue to the CNS 17–18. These analogues are potential agents for treating neurodegenerative disorders such as Alzheimer's disease.

### Carrier-mediated drug delivery:

This strategy takes advantage of the facilitative transport systems present in the brain endothelium. Active transport systems have been found for various substrates such as monosaccharides, monocarboxylic acids, amines, vitamins, hormones, purines, acidic and basic amino acids.

The cerebro vascular membranes are rich in facilitative carrier systems for both glucose and large neutral amino acids (LNAAs). The carrier system for glucose is highly specific for monosaccharides, rendering it useless for drug delivery. However, the LNAA carrier system is capable of transporting numerous endogenous as well as exogenous LNAAs with great structural variety 2,21. This characteristic has made exploitation of the LNAA carrier system an attractive strategy for CNS drug delivery.

The LNAA carrier system has been exploited to deliver L-3,4-dihydroxyphenalanine (levodopa), an endogenous precursor to dopamine, to the brain. Unlike dopamine, levodopa has a high affinity for the LNAA carrier system. After traversing the anti-luminal membrane of the cerebral endothelium, levodopa is decarboxylated to yield dopamine. The LNAA carrier system has also been exploited to deliver the antineoplastic agent melphalan into the brain.

### Receptor/vector-mediated drug delivery:

This strategy exploits several specific transcytosis systems that are actually meant for the extravasation of important nutrients and signaling molecules that cannot diffuse through the cerebromicrovasculature. These include systems for the transport of insulin, transferrin, and insulin like growth factor. Chimeric peptide delivery is one of such strategies, the principle of which lies in the coupling of a non-transportable peptide pharmaceutical to a transportable peptide or protein, which undergoes receptor mediated or absorptive mediated transcytosis through the BBB 22–23. For example, the non-transportable protein β-endorphin was linked to the transportable protein-cationized albumin via a disulfide linkage. This chimeric peptide was successfully transcytosed into the brain and enzymatically cleaved in the parenchyma by thiol reductase.

**Fig. 3:**
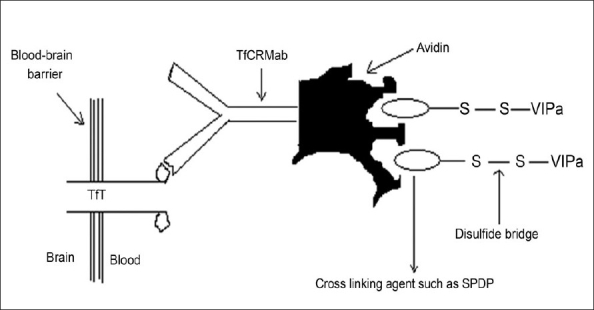
Transferrin transporter-based chemical peptide delivery. Peptide like drugs (VIPa) can be delivered to the brain by conjugating them to monoclonal antibodies (TfCRMab) by utilizing transporters such as transferrin transporter. TfT- transferring transporter; TfcRMab- antitransferrin receptor monoclonal antibody; SPDP- N-succinimidyl-3-(2-pyridyldithio)propionate; VIPa- vasoactive intestinal peptide analog.

Binding of the vector to its receptor on the luminal surface of brain capillary endothelial cells initiates endocytosis. Following exocytosis at the abluminal plasma membrane and release into brain interstitial space, the pharmacologically active moiety of the chimeric peptide may be released by enzymatic cleavage if a cleavable linkage between the vector and the drug is employed. The free peptide drug would then be able to interact with its specific target receptor on brain cells. A covalent conjugate of cationised albumin and the opioid peptide D-Ala-β-endorphin (DABE) was the first example of a chimeric opioid peptide to be investigated *in vitro*^24^ and *in vivo*^25^ with regard to its transport at the BBB. This chimeric peptide was linked by the disulfide based cross-linking agent, N-succinimidyl-3-(2-pyridyl-dithio) propionate (SPDP). The same technology has been applied to the vasoactive intestinal peptide analog (VIPa) (nontransportable pharmaceutical)^26^–^28^. This chimeric peptide consists of VIPa and a covalent conjugate of an antitransferrin receptor monoclonal antibody (mouse Mab OX26) and avidin vector (fig. 3).

The chimeric peptide technology has been applied to PNAs to achieve intracranial delivery of Polyamide (peptide) nucleic acids (PNAs) 29. This PNA can successfully be delivered to the murine brain parenchyma when covalently coupled to the OX-26 monoclonal antibody. This technique has been expanded to successfully deliver drug-loaded immunoliposomes to the brain 30. Immunoliposomes consist of liposomes coated with the inert polymer PEG conjugated to the OX-26 antitransferrin-receptor monoclonal antibody. Liposomes and proteins can be used as a cargo of membrane-permeable proteins for the improvement of their intracellular permeability^31^.

Nanoparticles have been used as cargo for peptides. PLGA nanoparticles are the most studied ones in this regard. These nanoparticles consist of a colloidal polymer particle of poly-butyl cyanoacrylate (PBCA) with the desired peptide adsorbed onto the surface and then coated with Polysorbate 80. Nanoparticles have been used as a vector for delivery of hexapeptide dalargin (an enkephalin analog)^32^–^33^. Drugs that have successfully been transported across the BBB with the nanoparticles include loperamide, tubocurarine and doxorubicin 34,35. The mechanism of nanoparticle transport has not yet been fully elucidated. The most probable transport pathway seems to be endocytosis by the blood capillary endothelial cells following adsorption of blood plasma components, most likely apolipoprotein E (apoE), after intravenous injection. Alternatively, transport may occur by transcytosis of the nanoparticles with drug across the endothelial cells. Percoating of nanoparticles with polysorbate led to adsorption of apoE and possibly other plasma components, which seem to be able to interact with the LDL receptors on the brain endothelial cells that could lead to their endocytosis. In addition to these processes, polysorbates seem to be able to inhibit efflux pump. This inhibition could contribute to the brain transport properties of the nanoparticles 36. Luca *et al,* found an increased permeability of PLGA nanoparticles when conjugated with five short peptides 37. Permeability of zidovudine and lamivudine across the BBB has been found to increase by 8-20 and 10-18 folds, respectively when administered as PBCA (polybutylcyanoacrylate) nanoparticles 38.

## DISRUPTING THE BBB

This invasive technique for enhanced CNS drug delivery involves the systemic administration of drugs in conjunction with transient BBB Disruption (BBBD). A variety of techniques that transiently disrupt the BBB have been investigated. However, many of these have been proved to be toxic and are not clinically useful. These include the infusion of solvents such as dimethyl sulfoxide or ethanol and metals such as aluminum, X-irradiation, and the induction of pathological conditions including hypertension, hypercapnia, hypoxia, or ischemia 3,39–41.

## Osmotic BBBD:

The most frequently applied clinical technique for achieving BBBD is the intracranial infusion of a hyperosmolar solution of mannitol 42,43. By infusing the solution directly into an artery that feeds the target area of the brain; it is somewhat possible to achieve localized BBBD and/or drug delivery 44. As the hyperosmolar solution flows through the cerebral capillaries, acute dehydration of endothelial cells results in cell shrinkage, which in turn widens the tight junctions connecting adjacent membranes. Subsequent rehydration in the presence of normal plasma leads to complete restoration of the BBB about 4 h. following treatment. An unfavourable toxic/therapeutic ratio often is observed with hyper osmotic BBBD. One reason for this is that this methodology results in a 25% increase in the permeability of the tumor microvasculature, in contrast to a 10-fold increase in the permeability of normal brain endothelium 45. Therefore the normal brain is left vulnerable to potentially neurotoxic chemotherapeutic agents.

## Biochemical BBBD:

These safer biochemical techniques selectively disrupt the intratumoral BBB while minimally altering the normal BBB 45. Selective opening of brain tumor capillaries, by the intracarotid infusion of leukotriene C4 was achieved without concomitant alteration of the adjacent BBB 46. It has been demonstrated in experimental animals that bradykinin, histamine and the synthetic bradykinin analog RMP-7 (receptor-mediated permeabilizer) infusion also selectively open the blood-tumor barrier. The biochemical mechanism has yet to be elucidated, but it has been established that the effect of the RMP-7 is mediated specifically through bradykinin B2 receptors.

## Ultrasound-induced disruption:

The possibility of enhanced drug delivery to the CNS by inducing hyperthermia has been investigated recently 47. Ultrasound induced mild hyperthermia, which can be controlled and localized to a small volume within the tissue, may offer promise 48. This technology is still in its infancy.

## ALTERNATIVE ROUTES FOR CNS DRUG DELIVERY

Changes at molecular levels and the mode of drug delivery may not always help in an increased penetration of drugs into the brain parenchyma. Certain methodologies have been proposed to enhance drug penetration into the brain, which are based on drug administration by alternative routes bypassing the cardiovascular system.

## Olfactory and Trigeminal pathways to the CNS:

**Fig. 4:**
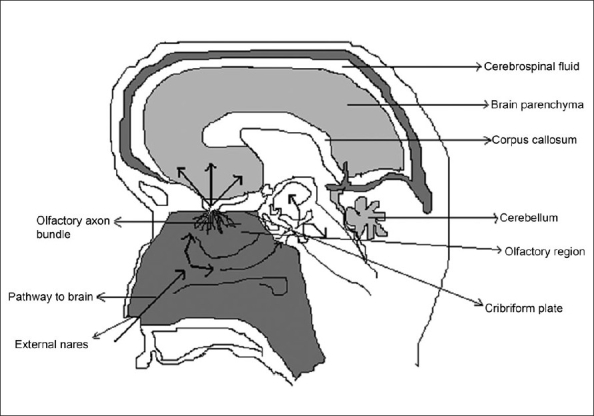
The Olfactory pathway between nose and central nervous system. Drugs can be delivered to the CNS by using olfactory route prevailing between nose and brain.

The neural pathway between the nasal mucosa and the brain provide a unique pathway for noninvasive delivery of therapeutic agents to the CNS 49–51 (fig. 4). The olfactory neural pathway provides both intraneuronal and extra neuronal pathways in to the brain. The intraneuronal pathway involves axonal transport and requires hours to days for drugs to reach different brain regions 52–56. The extra neuronal pathway probably relies on bulk transport through perineural channels, which deliver drug directly to the brain parenchymal tissue, to the cerebro spinal fluid (CSF), or to both. This extra neuronal pathway allows therapeutic agents to reach the CNS with in minutes. Intranasal delivery of agents to the CSF is not surprising as CSF normally drains along the olfactory axon bundles as they traverse the cribriform plate of the skull and approach the olfactory submucosa in the roof of the nasal cavity where the CSF is then diverted in to the nasal lymphatics 57,58

The Trigeminal neural pathway also may be involved in rapidly delivering protein therapeutic agents, such as insulin like growth factor-1 to the brain following intranasal administration. The trigeminal nerves innervating areas of the nasal cavity are responsible for most chemo-perception other than olfaction and sense diverse stimuli, including hot spices, formaldehyde, and other chemicals 59. The properties of small molecules, including size and lipophilicity, have been reported to affect delivery to the CNS following intranasal administration 60,61. The lower molecular weight and higher lipophilicity are favourable characteristics for rapid intranasal uptake of small molecules into the CNS.

There are limitations on the use of intranasal delivery as a means to bypass the BBB, including limitation on the concentrations achievable in different regions of the brain and spinal cord, which will vary with each agent. Delivery is expected to decrease with increasing molecular weight of the drug. Additionally, some therapeutic agents may be susceptible to partial degradation in the nasal mucosa or may cause irritation to the mucosa. Finally, nasal congestion from colds or allergies may interfere with this method of delivery.

## Intraventricular/intrathecal delivery:

Drugs can be infused intraventricularly using an Ommaya reservoir, a plastic reservoir implanted subcutaneously in the scalp and connected to the ventricles within the brain via an outlet catheter. Drug solutions can be subcutaneously injected into the implanted reservoir and delivered to the ventricles by manual compression of the reservoir through the scalp. This strategy has several advantages compared to vascular drug delivery. This route bypasses the BBB and results in high CSF drug concentrations. A smaller dose is enough and systemic toxicity can be greatly reduced 21. Drugs in the CSF face minimized protein binding and decreased enzymatic activity relative to drugs in plasma, leading to longer drug half-life in the CSF 62. However, this strategy has certain disadvantages, including a slow rate of drug distribution within the CSF and increase in intracranial pressure associated with fluid injection or infusion into small ventricular volumes, resulting in to high clinical incidence of haemorrhage, CSF leaks, neurotoxicity and CNS infections 22. CSF-brain barrier also limits the success of this approach.

## Interstitial delivery:

This route of administration bypasses BBB. High CNS drug concentrations can be obtained with minimal systemic exposure and toxicity. Intracranial drug concentrations can be sustained, which is crucial in the treatment of many neurodegenerative disorders and for the antitumor efficacy of many chemotherapeutic agents. Ommaya reservoir, infusaid pump, MiniMed PIMS system and Medtronic SynchroMed system are some of the systems, which have been developed for delivering drugs directly to the brain interstitium. Until recently the most widely used method has been the interstitial injection or infusion of drugs using an ommaya reservoir or implantable pump. The adaptation of the ommaya reservoir to achieve interstitial drug delivery simply involves placing the outlet catheter directly in the intracranial target area. This technique has often been applied to neurooncological patients in whom the outlet catheter is placed in the resection cavity following surgical de-bulking of a brain tumor. Chemotherapeutic agents can be periodically injected into the subcutaneous reservoir and then delivered directly to the tumor bed. This technique, however, does not achieve truly continuous drug delivery.

The ommaya reservoir or infusion pumps have thus far been used in various clinical trials with brain tumor patients to interstitially deliver the chemotherapeutic agents BCNU or its analogs, methotrexate, adriamycin, bleomycin, fluodeoxyuridine, cisplatin, and interleukin 2(IL-2). In most of these studies the intratumoral drug concentrations were often high, and the side effects of the therapy were mild. The success of these techniques is limited by catheter clogging or blocking by tissue debris, inadequate distribution throughout the tumor, and a high degree of burden to the patient.

## Biotechnological approaches:

Achieving interstitial drug delivery using biological tissues is another promising technique. It involves implanting into the brain, a tissue that naturally secretes a desired therapeutic agent. Transplantation of embryonic dopamine-releasing neurons is an attractive therapeutic strategy because these cells demonstrate good post-transplantation survival and growth characteristics in animal models 63. However, lack of neovascular innervation limits this strategy. With out rapid neovascularization, implanted solid grafts undergo irreparable ischemic injury, leading to cell death.

Gene therapy has also been attempted to deliver drugs to the CNS. Prior to implantation, cells will be genetically modified to synthesize and release specific therapeutic agents. The therapeutic potential of this technique in the treatment of brain tumor was demonstrated. The utility of non-neuronal cells for therapeutic protein delivery to the CNS has been reviewed recently 64. The survival of foreign tissue grafts may be improved by advancements in techniques for culturing distinct cell types. Co-grafted cells engineered to release neurotropic factors with cells engineered to release therapeutic proteins may enhance the survival and development of foreign tissue 65. Direct application of protein-based therapeutics to the brain could soon include variations of diphtheria toxin to combat refractory gliobastomas and engineered anti-apoptotic factor (FNK) with powerful cytoprotective activity, to protect against ischemia 66,67. As for neurodegeneration, one seemingly attractive new therapy has been the use of growth factors, such as glial-derived neurotrophic factor (GDNF) as a potential means of reducing the depletion of certain key population of cells lost in Alzheimer's or Parkinson's diseases 68,69.

**Fig. 5:**
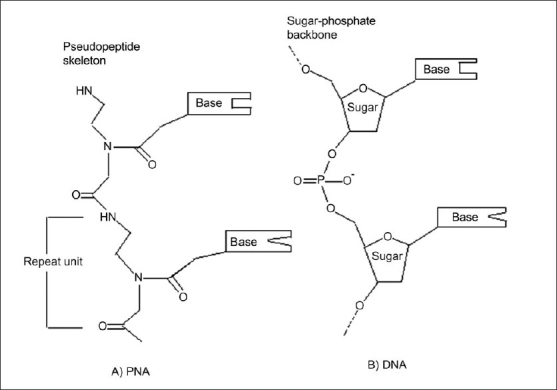
Structural Similarity between PNA and DNA oligomers. The molecular mimicry of DNA into Polyamide (peptide) nucleic acids (PNAs) has helped the delivery of the chimeric peptide to intra cranial delivery.

Antisense drug delivery is another recent technology in CNS drug delivery. Peptide nucleic acids (PNAs) (fig. 5) are antisense oligonucleotides containing a polypeptide backbone. Receptor mediated transcytosis has been exploited to promote PNA delivery to the CNS. For example, the attachment of PNAs to the anti-transferrin (OX26) receptor antibodies has been shown to increase the brain uptake of the PNAs, with out loss of the ability of the PNAs to hybridize to target mRN 70.
